# Diiodidobis(*N*,*N*,*N*′,*N*′-tetra­methyl­thio­urea-κ*S*)cadmium(II)

**DOI:** 10.1107/S1600536810028114

**Published:** 2010-07-17

**Authors:** Sidra Nawaz, Sana Sadaf, Mohammed Fettouhi, Atif Fazal, Saeed Ahmad

**Affiliations:** a Department of Chemistry, University of Engineering and Technology, Lahore 54890, Pakistan; b Department of Chemistry, King Fahd University of Petroleum and Minerals, Dhahran 31261, Saudi Arabia

## Abstract

In the title compound, [CdI_2_(C_5_H_12_N_2_S)_2_], the Cd^II^ ion is located on a twofold rotation axis and is coordinated in a distorted tetra­hedral mode by two iodide ions and by two tetra­methyl­thio­urea (tmtu) ligands through their S atoms. The crystal structure is stabilized by C—H⋯N and C—H⋯S hydrogen bonds.

## Related literature

For background to thio­urea complexes of group 12 elements, see: Ahmad *et al.* (2009 ▶); Bell *et al.* (2001 ▶, 2004 ▶); Lobana *et al.* (2008 ▶); Marcos *et al.* (1998 ▶); Matsunaga *et al.* (2005 ▶); Moloto *et al.* (2003 ▶); Wazeer *et al.* (2007 ▶). The structure of the title compound is isotypic with [Cd(tmtu)_2_Br_2_] (Nawaz *et al.*, 2010*a* ▶) and [Hg(tmtu)_2_Cl_2_] (Nawaz *et al.*, 2010*b* ▶).
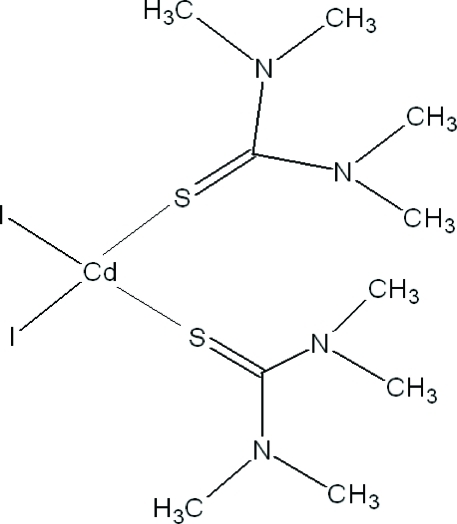


## Experimental

### 

#### Crystal data

[CdI_2_(C_5_H_12_N_2_S)_2_]
*M* *_r_* = 630.65
Monoclinic, 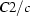

*a* = 18.985 (5) Å
*b* = 10.395 (3) Å
*c* = 13.719 (4) Å
β = 130.740 (4)°
*V* = 2051.4 (9) Å^3^
*Z* = 4
Mo *K*α radiation
μ = 4.27 mm^−1^
*T* = 294 K
0.33 × 0.22 × 0.20 mm

#### Data collection

Bruker SMART APEX area-detector diffractometer
Absorption correction: multi-scan (*SADABS*; Sheldrick, 1996 ▶) *T*_min_ = 0.333, *T*_max_ = 0.482
13642 measured reflections
2557 independent reflections
2235 reflections with *I* > 2σ(*I*)
*R*_int_ = 0.022

#### Refinement

*R*[*F* ^2^ > 2σ(*F* ^2^)] = 0.023
*wR*(*F* ^2^) = 0.054
*S* = 1.04
2557 reflections
91 parameters
H-atom parameters constrained
Δρ_max_ = 0.67 e Å^−3^
Δρ_min_ = −0.58 e Å^−3^

### 

Data collection: *SMART* (Bruker, 2008 ▶); cell refinement: *SAINT* (Bruker, 2008 ▶); data reduction: *SAINT*; program(s) used to solve structure: *SHELXS97* (Sheldrick, 2008 ▶); program(s) used to refine structure: *SHELXL97* (Sheldrick, 2008 ▶); molecular graphics: *SHELXTL* (Sheldrick, 2008 ▶); software used to prepare material for publication: *SHELXTL*.

## Supplementary Material

Crystal structure: contains datablocks I, global. DOI: 10.1107/S1600536810028114/wm2373sup1.cif

Structure factors: contains datablocks I. DOI: 10.1107/S1600536810028114/wm2373Isup2.hkl

Additional supplementary materials:  crystallographic information; 3D view; checkCIF report

## Figures and Tables

**Table 1:** Hydrogen-bond geometry (Å, °)

*D*—H⋯*A*	*D*—H	H⋯*A*	*D*⋯*A*	*D*—H⋯*A*
C2—H2*A*⋯N2	0.96	2.51	2.859 (6)	101
C4—H4*A*⋯N1	0.96	2.52	2.853 (5)	100
C5—H5*A*⋯S1	0.96	2.66	3.026 (5)	103

**Fig. 1.**
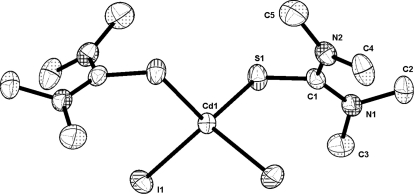
The molecular structure of title compound with atomic numbering scheme. Displacement ellipsoids drawn at the 30% probability level. H-atoms were omitted for clarity.

## supplementary crystallographic information

### Comment

A considerable amount of work has been done in recent years on the synthesis and characterization of cadmium(II) and mercury(II) complexes of thiourea type ligands due to their variable binding modes and because of the their importance in biological systems (Ahmad *et al.*, 2009; Bell *et al.*, 2001, 2004; Lobana *et al.*, 2008; Marcos *et al.*, 1998; Matsunaga *et al.*, 2005; Moloto *et al.*, 2003; Wazeer *et al.*, 2007). Cadmium(II) complexes with thiones possess a variety of structures ranging from four- to six-coordinate species with tetrahedral and octahedral environments for the Cd^II^ atom, respectively. In some cases, these units further aggregate to form polymeric structures (Bell *et al.*, 2001, 2004; Lobana *et al.*, 2008; Matsunaga *et al.*, 2005; Moloto, *et al.*, 2003; Wazeer *et al.*, 2007). We report here the crystal structure of a cadmium(II) iodide complex with tetramethylthiourea (tmtu).

In the title complex, the cadmium atom is bonded to two I^-^ ions and to two tetramethylthiourea ligands through the sulfur atoms in a distorted tetrahedral mode (Fig. 1). The compound is isotypic with [Cd(tmtu)_2_Br_2_] (Nawaz *et al.*, 2010*a*) and [Hg(tmtu)_2_Cl_2_] (Nawaz *et al.*, 2010*b*).

For a more detailed description of the structure, see: Nawaz *et al.* (2010*a*).

### Experimental

To 0.37 g (1.0 mmol) cadmium(II) iodide in 10 ml water was added to two equivalents of tetramethylthiourea in 15 ml methanol. A clear solution was obtained that was stirred for 30 minutes. The colorless solution was filtered and the filtrate was kept at room temperature for crystallization. As a result, a white crystalline product was obtained, that was washed with methanol and dried.

### Refinement

H atoms were placed in calculated positions with a C—H distance of 0.96 Å and *U*_iso_(H) = 1.5 *U*_eq_(C).

### Crystal data

**Table:**

[CdI_2_(C_5_H_12_N_2_S)_2_]	*F*(000) = 1192
*M**_r_* = 630.65	*D*_x_ = 2.042 Mg m^−^^3^
Monoclinic, *C*2/*c*	Mo *K*α radiation, λ = 0.71073 Å
Hall symbol: -C 2yc	Cell parameters from 13642 reflections
*a* = 18.985 (5) Å	θ = 2.4–28.3°
*b* = 10.395 (3) Å	µ = 4.27 mm^−^^1^
*c* = 13.719 (4) Å	*T* = 294 K
β = 130.740 (4)°	Block, colorless
*V* = 2051.4 (9) Å^3^	0.33 × 0.22 × 0.20 mm
*Z* = 4	

### Data collection

**Table:**

Bruker SMART APEX area-detector diffractometer	2557 independent reflections
Radiation source: normal-focus sealed tube	2235 reflections with *I* > 2σ(*I*)
graphite	*R*_int_ = 0.022
ω scans	θ_max_ = 28.3°, θ_min_ = 2.4°
Absorption correction: multi-scan (*SADABS*; Sheldrick, 1996)	*h* = −25→25
*T*_min_ = 0.333, *T*_max_ = 0.482	*k* = −13→13
13642 measured reflections	*l* = −18→18

### Refinement

**Table:**

Refinement on *F*^2^	Primary atom site location: structure-invariant direct methods
Least-squares matrix: full	Secondary atom site location: difference Fourier map
*R*[*F*^2^ > 2σ(*F*^2^)] = 0.023	Hydrogen site location: inferred from neighbouring sites
*wR*(*F*^2^) = 0.054	H-atom parameters constrained
*S* = 1.04	*w* = 1/[σ^2^(*F*_o_^2^) + (0.0229*P*)^2^ + 2.9795*P*] where *P* = (*F*_o_^2^ + 2*F*_c_^2^)/3
2557 reflections	(Δ/σ)_max_ = 0.002
91 parameters	Δρ_max_ = 0.67 e Å^−^^3^
0 restraints	Δρ_min_ = −0.58 e Å^−^^3^

### Special details

**Table:**

Geometry. All e.s.d.'s (except the e.s.d. in the dihedral angle between two l.s. planes) are estimated using the full covariance matrix. The cell e.s.d.'s are taken into account individually in the estimation of e.s.d.'s in distances, angles and torsion angles; correlations between e.s.d.'s in cell parameters are only used when they are defined by crystal symmetry. An approximate (isotropic) treatment of cell e.s.d.'s is used for estimating e.s.d.'s involving l.s. planes.
Refinement. Refinement of *F*^2^ against ALL reflections. The weighted *R*-factor *wR* and goodness of fit *S* are based on *F*^2^, conventional *R*-factors *R* are based on *F*, with *F* set to zero for negative *F*^2^. The threshold expression of *F*^2^ > σ(*F*^2^) is used only for calculating *R*-factors(gt) *etc*. and is not relevant to the choice of reflections for refinement. *R*-factors based on *F*^2^ are statistically about twice as large as those based on *F*, and *R*- factors based on ALL data will be even larger.

### Fractional atomic coordinates and isotropic or equivalent isotropic displacement parameters (Å^2^)

**Table:**

	*x*	*y*	*z*	*U*_iso_*/*U*_eq_	
Cd1	1.0000	0.71325 (3)	0.2500	0.04562 (8)	
I1	1.155686 (14)	0.56794 (2)	0.35345 (2)	0.06074 (8)	
S1	1.03756 (5)	0.84000 (9)	0.43960 (7)	0.05829 (19)	
N1	0.92464 (19)	0.7668 (2)	0.4801 (3)	0.0568 (6)	
N2	0.85712 (18)	0.8932 (3)	0.3011 (2)	0.0569 (6)	
C1	0.93150 (19)	0.8328 (3)	0.4031 (3)	0.0462 (6)	
C2	0.8634 (3)	0.8067 (4)	0.5045 (4)	0.0777 (10)	
H2A	0.8343	0.8869	0.4619	0.116*	
H2B	0.8992	0.8168	0.5954	0.116*	
H2C	0.8165	0.7423	0.4722	0.116*	
C3	0.9920 (3)	0.6671 (4)	0.5676 (4)	0.0870 (12)	
H3A	1.0154	0.6268	0.5308	0.130*	
H3B	0.9622	0.6037	0.5808	0.130*	
H3C	1.0425	0.7054	0.6486	0.130*	
C4	0.7619 (2)	0.8470 (4)	0.2310 (4)	0.0808 (11)	
H4A	0.7637	0.7632	0.2621	0.121*	
H4B	0.7299	0.8422	0.1408	0.121*	
H4C	0.7297	0.9054	0.2444	0.121*	
C5	0.8660 (3)	0.9963 (4)	0.2369 (4)	0.0840 (11)	
H5A	0.9259	1.0360	0.2972	0.126*	
H5B	0.8184	1.0594	0.2048	0.126*	
H5C	0.8594	0.9610	0.1666	0.126*	

### Atomic displacement parameters (Å^2^)

**Table:**

	*U*^11^	*U*^22^	*U*^33^	*U*^12^	*U*^13^	*U*^23^
Cd1	0.04989 (15)	0.04876 (16)	0.04920 (15)	0.000	0.03717 (13)	0.000
I1	0.05619 (12)	0.06315 (14)	0.06036 (13)	0.01437 (9)	0.03692 (11)	0.00562 (9)
S1	0.0509 (4)	0.0769 (5)	0.0529 (4)	−0.0122 (4)	0.0365 (3)	−0.0185 (4)
N1	0.0677 (15)	0.0552 (14)	0.0648 (15)	0.0083 (12)	0.0508 (14)	0.0065 (12)
N2	0.0576 (14)	0.0651 (15)	0.0490 (13)	0.0072 (12)	0.0352 (12)	−0.0014 (11)
C1	0.0517 (14)	0.0473 (14)	0.0470 (13)	−0.0003 (11)	0.0355 (12)	−0.0087 (11)
C2	0.092 (3)	0.090 (3)	0.091 (3)	0.005 (2)	0.077 (2)	0.003 (2)
C3	0.109 (3)	0.067 (2)	0.103 (3)	0.025 (2)	0.077 (3)	0.030 (2)
C4	0.0502 (17)	0.118 (3)	0.068 (2)	0.0085 (19)	0.0360 (17)	−0.014 (2)
C5	0.102 (3)	0.081 (3)	0.064 (2)	0.019 (2)	0.052 (2)	0.0211 (19)

### Geometric parameters (Å, °)

**Table:**

Cd1—S1	2.5670 (9)	C2—H2B	0.9600
Cd1—S1^i^	2.5670 (10)	C2—H2C	0.9600
Cd1—I1^i^	2.7489 (7)	C3—H3A	0.9600
Cd1—I1	2.7489 (7)	C3—H3B	0.9600
S1—C1	1.731 (3)	C3—H3C	0.9600
N1—C1	1.335 (4)	C4—H4A	0.9600
N1—C2	1.465 (4)	C4—H4B	0.9600
N1—C3	1.466 (4)	C4—H4C	0.9600
N2—C1	1.330 (4)	C5—H5A	0.9600
N2—C5	1.464 (5)	C5—H5B	0.9600
N2—C4	1.468 (4)	C5—H5C	0.9600
C2—H2A	0.9600		
			
S1—Cd1—S1^i^	118.23 (5)	H2A—C2—H2C	109.5
S1—Cd1—I1^i^	107.41 (2)	H2B—C2—H2C	109.5
S1^i^—Cd1—I1^i^	105.36 (2)	N1—C3—H3A	109.5
S1—Cd1—I1	105.36 (2)	N1—C3—H3B	109.5
S1^i^—Cd1—I1	107.41 (2)	H3A—C3—H3B	109.5
I1^i^—Cd1—I1	113.34 (3)	N1—C3—H3C	109.5
C1—S1—Cd1	100.59 (9)	H3A—C3—H3C	109.5
C1—N1—C2	122.5 (3)	H3B—C3—H3C	109.5
C1—N1—C3	121.9 (3)	N2—C4—H4A	109.5
C2—N1—C3	114.3 (3)	N2—C4—H4B	109.5
C1—N2—C5	121.3 (3)	H4A—C4—H4B	109.5
C1—N2—C4	122.9 (3)	N2—C4—H4C	109.5
C5—N2—C4	114.9 (3)	H4A—C4—H4C	109.5
N2—C1—N1	119.4 (3)	H4B—C4—H4C	109.5
N2—C1—S1	121.3 (2)	N2—C5—H5A	109.5
N1—C1—S1	119.3 (2)	N2—C5—H5B	109.5
N1—C2—H2A	109.5	H5A—C5—H5B	109.5
N1—C2—H2B	109.5	N2—C5—H5C	109.5
H2A—C2—H2B	109.5	H5A—C5—H5C	109.5
N1—C2—H2C	109.5	H5B—C5—H5C	109.5

Symmetry codes: (i) −*x*+2, *y*, −*z*+1/2.

### Hydrogen-bond geometry (Å, °)

**Table:**

*D*—H···*A*	*D*—H	H···*A*	*D*···*A*	*D*—H···*A*
C2—H2A···N2	0.96	2.51	2.859 (6)	101
C4—H4A···N1	0.96	2.52	2.853 (5)	100
C5—H5A···S1	0.96	2.66	3.026 (5)	103
